# Clinical text mining of the performance status and progression-free survival to facilitate data collection in cancer research: an exploratory study

**DOI:** 10.1016/j.esmorw.2024.100059

**Published:** 2024-08-13

**Authors:** L. Lin, M. Singer-van den Hout, L.F.A. Wessels, A.J. de Langen, J.H. Beijnen, A.D.R. Huitema

**Affiliations:** 1 Department of Pharmacy & Pharmacology; 2 Department of Biometrics, The Netherlands Cancer Institute-Antoni van Leeuwenhoek Hospital, Amsterdam; 3 Division of Molecular Carcinogenesis, The Netherlands Cancer Institute-Antoni van Leeuwenhoek Hospital, Amsterdam; 4 Oncode Institute, Utrecht; 5 Department of Electrical Engineering, Mathematics and Computer Science, Delft University of Technology, Delft; 6 Department of Thoracic Oncology, The Netherlands Cancer Institute-Antoni van Leeuwenhoek Hospital, Amsterdam; 7 Department of Pharmaceutical Sciences, Utrecht University, Utrecht; 8 Department of Pharmacology, Princess Máxima Center for Pediatric Oncology, Utrecht; 9 Department of Clinical Pharmacy, University Medical Center Utrecht, Utrecht University, Utrecht, The Netherlands

**Keywords:** text mining, natural language processing, real-world data, progression-free survival, performance status

## Abstract

**Background:**

Modern electronic medical records (EMRs) contain a valuable amount of data. These data can be unlocked for research by manual data collection, which is highly labor intensive. Therefore, we explored whether automated text mining (TM) could be used to extract the performance status (PS) and progression-free survival (PFS) in a cohort of 328 non-small-cell lung cancer patients.

**Materials and methods:**

Unstructured Dutch text data were derived from different EMR fields containing mainly information recorded during outpatient visits. A rule-based TM approach using regular expressions was used to extract PS and PFS in the R programming language. For PS, quantitative evaluation metrics, such as the weighted F1-score, were used to determine the accuracy of the TM-extracted data. For PFS, the median PFS was compared between the two approaches using the Kaplan–Meier method. In addition, the C-index was determined.

**Results:**

A PS was obtained for 196 patients (60%) using the TM approach. In 189 (96%) patients, the TM-curated PS matched the manually curated PS. The weighted F1-score was 96.5%. The median PFS was 7.42 months for the manually curated data (*n* = 328) and 8.00 months for the TM-curated data (*n* = 301). The C-index was 0.916.

**Conclusions:**

The developed TM approach is able to extract PS and PFS from the EMR with a very good performance. Therefore, this approach increases the efficiency of reliable data collection from EMRs, facilitating the use of real-world data (RWD) in clinical research.

## Highlights

• Manual data collection from EMRs is highly labor intensive.
• TM techniques can increase the efficiency of data collection.
• TM tools are essential to advance artificial intelligence models using RWD.

## Introduction

Over the years, there has been an increasing interest in the value of real-world evidence.^1^ Electronic medical records (EMRs) are one of the sources containing a valuable amount of real-world data (RWD). These RWD are suitable for addressing clinical and policy-related questions.^2^

A minor part of all the data in EMRs is stored as structured data, e.g. diagnosis codes and laboratory values. Therefore, the majority of data in EMRs are stored as free text in an unstructured manner.^3^ Consequently, clinical researchers often have to collect the data of interest manually, which is highly labor intensive.

Tools to facilitate data extraction from EMRs include text mining (TM) and natural language processing (NLP) techniques. Within NLP techniques, the rule-based NLP approach and the statistical NLP approach can be distinguished. The first approach uses expert knowledge to define rules. In contrast, the second approach learns to carry out its task by applying machine learning algorithms to training data.^4^ These tools can greatly improve the efficiency of data collection from EMRs, as was shown in a study comparing a commercial TM software tool to manual data collection.^5^

In addition to commercial tools, open-source software systems are available in different programming languages, such as Python, Java and R.^4^ As clinical researchers frequently use R to carry out statistical analyses, this programming language is more accessible to them than other programming languages. Therefore, we explored whether an automated rule-based TM approach designed in R could be used to extract valuable data from unstructured text from EMRs. In this exploratory study, we attempted to extract the performance status (PS) and progression-free survival (PFS) of a cohort of non-small-cell lung cancer (NSCLC) patients. These two data types are not regularly stored in a structured manner and are very important in clinical research determining treatment effectiveness.

## Materials and methods

This exploratory study was conducted at the Netherlands Cancer Institute—Antoni van Leeuwenhoek hospital (NKI-AvL), Amsterdam, The Netherlands. At our institute, the EMR HiX (ChipSoft, Amsterdam, The Netherlands) is used. We tried to extract PS and PFS in a patient population consisting of 328 NSCLC patients treated with post-first-line osimertinib. The PS and PFS data were already manually collected for another study. Therefore, the current study used these data to determine the accuracy of the TM-extracted data.^6^ The PS is a measure indicating patients’ ability to carry out daily activities. PFS was defined as the time from osimertinib treatment initiation until the first signs of disease progression by radiology or clinical signs, or death by any cause in the absence of disease progression.^6^^,^^7^

### Developed text mining approach

**Figure 1:**
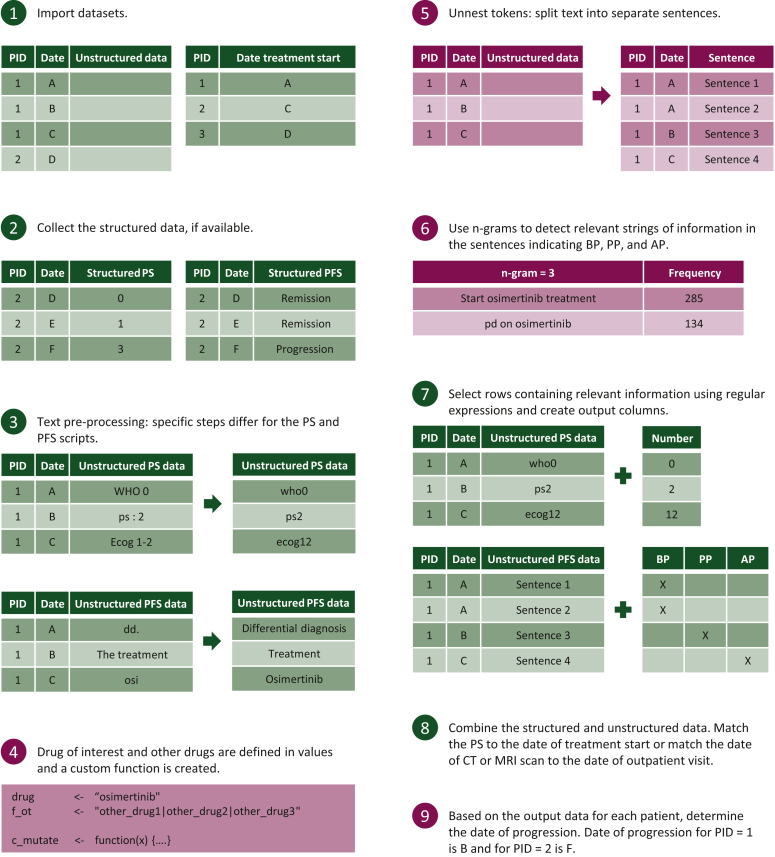
**Overviewof the steps taken to extract PS and PFS.** The green steps are applicable for both the PS and PFS scripts. Burgundy steps are only applicable for the PFS script. AP, after progression; BP, before progression; CT, computed tomography; MRI, magnetic resonance imaging; PID, patient identifier; PS, performance status; PFS, progression-free survival; PP, possible progression.

The developed TM method used a systematic approach, which is summarized in Figure 1. To keep the TM approach accessible to clinical researchers, we used a limited number of functions from well-known packages. This resulted in R scripts mainly using functions from base R, tidyverse and tidytext (Supplementary Material, available at https://doi.org/10.1016/j.esmorw.2024.100059).

#### Step 1: Data selection from EMRs

We identified unstructured fields in the EMR containing relevant information regarding PS and PFS. Several patients were manually screened to determine the relevant fields. Initially, we only considered data from outpatient visits. Later, data during hospital admissions and data documented in small notes were also taken into consideration.

Then, we exported the unstructured texts in these relevant fields from the EMRs into separate Excel files for PS and PFS. These files were used as input files in R. The layout of these files is described in the Supplementary Material, available at https://doi.org/10.1016/j.esmorw.2024.100059. Our TM approach used a second input file with unique patient identifiers and the date of osimertinib treatment initiation. At our institute, the date of treatment initiation is structurally stored in the dispensing data from the hospital pharmacy. These data were exported in a second input file in R. Using this second file, all data before the initiation of osimertinib treatment of each patient were removed from the PFS input file.

#### Step 2: Extracting structured data

Our EMR has the functionality to store PSs as structured data similar to the documentation of laboratory values. Similarly, our EMR has the functionality to document whether a patient is in remission or has progression as a closed question during each outpatient visit. These data were collected, but represented only a small proportion of the total amount of data.

#### Step 3: Text pre-processing

We incorporated text pre-processing steps to clean and prepare the unstructured text for data extraction.^8^ The required pre-processing steps varied depending on the data type. PSs were documented in slightly different formats. Defining all possible formats would result in an inefficient TM approach. Removing uppercases, spaces and punctuation marks (except periods and commas, as these separate sentences and part of sentences) minimized the number of possible formats describing PS.

For PFS, the pre-processing steps were selected to facilitate correct tokenization, i.e. breaking up clinical text into separate sentences, and data extraction in later steps. For example, abbreviations with periods interfered with the tokenization of unstructured clinical text. Therefore, frequently occurring abbreviations were replaced with the full words. We considered removing stop words, which are extremely common words that are not useful in text analysis.^8^ The Dutch stop word set from the lsa package contained words equivalent to ‘no’ and ‘maybe’. Removing these words would change the meaning of sentences in the context of progression. Therefore, we did not use stop word sets. Only the stop words ‘de’ and ‘het’, which are equivalent to ‘the’ in English, were removed. In addition, osimertinib and crizotinib were often shortened in the EMR to ‘osi’ and ‘crizo’. These were also replaced by their full name to facilitate the use of regular expressions in step 7.

#### Step 4: Define the drug of interest (only relevant for PFS)

The drug of interest was defined, which can include the generic and brand name. Drugs that were used after progression on osimertinib were also defined. This implementation allows for changing the drug of interest without changing the code in step 7. In addition, a custom function was created, which is used in step 7 to define whether progression has occurred.

#### Step 5: Tokenization (only relevant for PFS)

The clinical text was tokenized into individual sentences enabling the analysis of each individual sentence. Clinical text often lacked punctuation, as health care provides often used whitespace and new lines to indicate new sentences. Therefore, the built-in option to tokenize sentences in the tidytext package was not suitable. Instead, a custom tokenizing function was defined.

#### Step 6: Detect relevant information (only relevant for PFS)

Using expert knowledge only is often not enough to obtain all relevant text strings. Therefore, sentences were further tokenized into small groups of successive words, called n-grams, to detect relevant text strings. For PFS, n-grams indicating the time before progression (BP), around progression (PP) and after progression (AP) were determined. Ideally, the aim is to keep the interval of PP as small as possible for an accurate determination of the time of progression.

#### Step 7: Extracting relevant information

A rule-based NLP approach that used regular expressions was employed. Regular expressions are concise and flexible tools to describe certain text strings.^9^ These were used to define the relevant text strings detected in the previous step. A few examples of these regular expressions are shown in the Supplementary Material, available at https://doi.org/10.1016/j.esmorw.2024.100059. We chose this approach so that clinical researchers themselves can define relevant information. In addition, it is clear what information will be found and what information will not be found depending on the definition of the regular expressions.

For PS, a single regular expression was defined to search for words indicating PS (e.g. ‘ps’, ‘who’, ‘ecog’) followed by at most two numbers that can indicate a PS. For PFS, strings of text indicating BP, PP and AP were collected. Examples of regular expressions indicating these phases during treatment are provided in the Supplementary Material, available at https://doi.org/10.1016/j.esmorw.2024.100059, with additional explanation of the code construction. For PFS, we provided a shorter and modified version of the actual script for better readability as repetition of the same code was present for different regular expressions. The regular expressions presented in the R scripts are in Dutch accompanied by English translations.

#### Step 8: Combining structured and unstructured data

The structured and unstructured PSs from step 2 and step 7 were combined. The PS closest to treatment initiation within 30 days was matched for each patient. If no PS before treatment initiation was matched, a PS up to 14 days after treatment initiation was also allowed.

In the PFS script, the date of computed tomography (CT) or magnetic resonance imaging (MRI) scan was matched to the date of data entry in the original script. These scans often occur a few days before outpatient visits. Therefore, in case of progression, the scan date was more accurate than the date of the outpatient visit. Due to data migration and the use of different date formats, the code matching the date of CT or MRI scan to the date of outpatient visit was too specific for our institute. Therefore, this part is omitted in the modified PFS script. However, a similar code used to match PS to the date of treatment initiation can be used as a guide to match the dates of CT or MRI scans.

#### Step 9: Determine the date of progression (only relevant for PFS)

For the PS script, the output file contains the PS matched to the date of treatment initiation. For the PFS script, the output file contains a chronological overview for each patient describing whether progression occurred at a certain time point. Based on this overview, the date of progression was determined for each individual patient.

### Statistical analysis

After step 9, the TM-collected data were compared to the manually collected data. For PS, quantitative evaluation metrics were used to determine class-wise performance scores for each possible value for PS. In addition, the weighted F1-score was determined to ascertain the overall performance of the TM approach for PS. Precision, recall and the F1-score could vary from 0% to 100%. A score closer to 100% indicates a better performance of the TM approach. In addition, in the case of discrepancies between the TM approach and manual data collection, the cause of the discrepancy was determined.

For PFS, the median PFS for both approaches was compared using the Kaplan–Meier method and the 95% confidence intervals (CIs) were calculated. In addition, the C-index, which describes the performance of the TM approach, was determined. The C-index can vary from 0 to 1, in which scores closer to 1 indicate better performances. Furthermore, progression dates determined by the two methods were visually compared using a scatterplot. The ratio between the TM and the manually curated PFS was determined. Individuals with a ratio >3 or <0.33 were analyzed to determine the cause of discrepancy. Scripts were coded using R version 4.2.1 (R Foundation for Statistical Computing, Vienna, Austria).

## Results

Patient characteristics can be found in a previously published paper.^6^ At the initiation of osimertinib treatment, 90, 163, 51 and 22 patients had PSs of 0, 1, 2, 3 and 4, respectively. For two patients, no PS was available at treatment initiation. The median duration of follow-up was 27.8 months. At the time of data cut-off, disease progression occurred in 286 patients.

### Performance status

PS was documented 1265 times in a structured manner across 117 patients in the EMR. The developed TM approach obtained an additional 2897 instances of PS across 306 patients.

**Figure 2:**
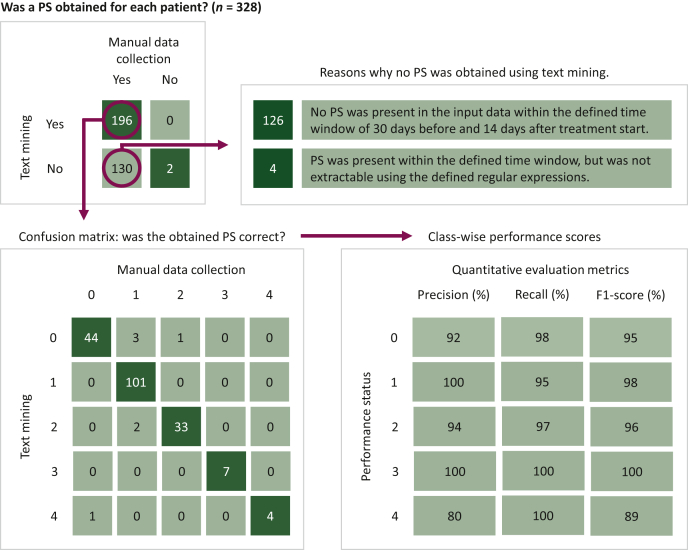
**Flow chart of collected performance status (PS) using manual data collection and the text mining approach including class-wise performance scores.** PS, performance status.

The TM approach found a matched PS for 196 patients (60%). Figure 2 depicts the confusion matrix comparing the TM-curated and manually curated PSs including the quantitative evaluation metrics. Class-wise precision, recall and F1-scores varied from 80 to 100. The weighted F1-score was 96.5%.

In 189 patients (96%), the TM-curated PS was in accordance with the manually curated PS. In seven patients (4%), the matched PS deviated from the manually collected PS. In six patients, the PS closest to treatment initiation was not representative of the PS at the moment of treatment initiation. Between these dates, the patient’s condition significantly improved or worsened. These cases were observed during manual data collection and an accurate PS was obtained otherwise. In the remaining case, the manually curated PS was faulty and the TM-curated PS was actually preferred.

For 130 patients (40%), the TM approach did not obtain a PS. In 126 patients (95%), no PS was present around the time of treatment initiation in the EMR. In our previous study, these PSs were obtained otherwise. The correct PS was present in the EMR for four patients (3%). In one patient, the number 0 was misspelled with the letter O (i.e. ‘WHO O’). In the other patients, additional text in between the word describing the PS and the actual score (e.g. ‘PS is 2’) caused the failed data extractions.

### Progression-free survival

**Figure 3:**
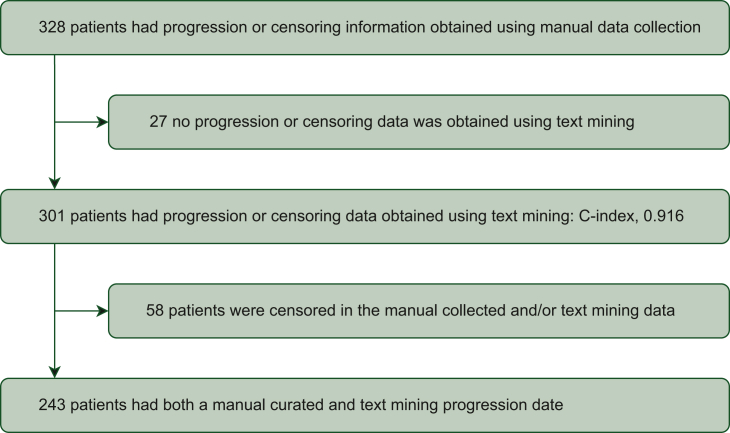
Flow chart of the collected date of progression using manual data collection and the text mining approach including the C-index describing the performance.

**Figure 4:**
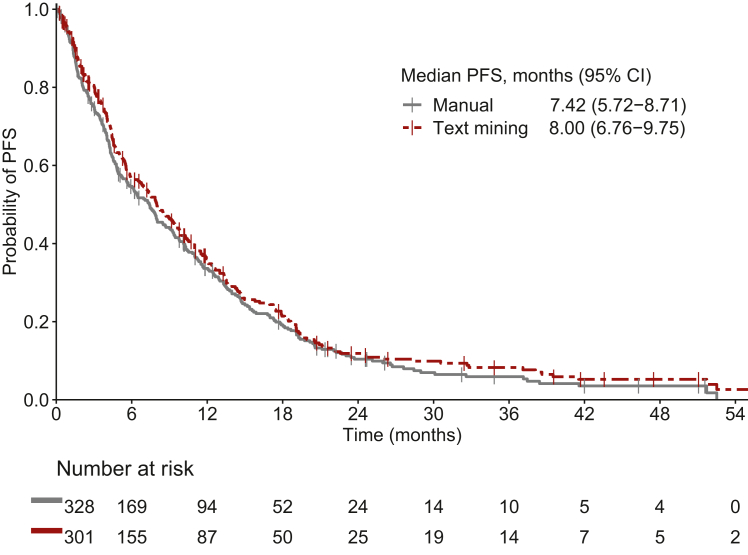
**Kaplan–Meier curves for PFS using manual data collection and the text mining approach.** CI, confidence interval; PFS, progression-free survival.

The number of patients included in each analysis for PFS is depicted in Figure 3. The Kaplan–Meier curves for the manually collected and TM-extracted PFS are shown in Figure 4. The median PFS for the manually curated data was 7.42 months (95% CI 5.72-8.71 months) (*n* = 328) and the median PFS for the TM approach was 8.00 months (95% CI 6.76-9.75 months) (*n* = 301).

The TM approach did not obtain a date of progression or date of censoring for 27 patients (8%). In 12 patients (4%), progression was described in a PDF file. For nine patients (3%), no follow-up data were available in the input file due to death shortly after treatment initiation, as the date of death was not present in the input file. (Leaving out the date of death in the input file was an intentional choice to prevent incorrect assumptions. In these cases, we believe that manual data collection is more appropriate.) In the remaining six patients, the time of progression was described in the EMR, but in a vague manner. For example, ‘growth of a metastasis’ was described in combination with ‘a puncture to determine the mechanism of resistance’ for one patient. These specific cases were not defined in step 7.

The C-index was determined using the data of 301 patients with both a manually collected and TM progression. This resulted in a C-index of 0.916.

**Figure 5:**
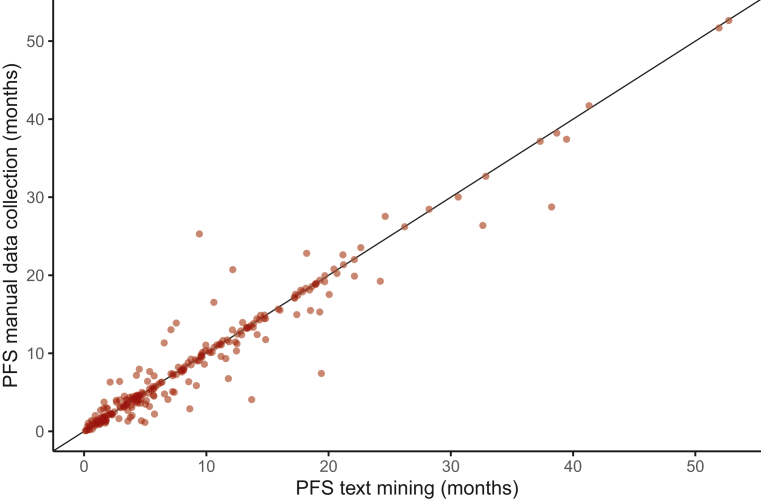
**Scatterplot showing PFS in months for the text mining approach versus manual data collection.** PFS, progression-free survival.

A total of 243 patients had both a TM and manually curated date of progression. Individual differences between the two approaches for these patients are depicted in Figure 5. A ratio >3 between the TM and manually curated PFS was observed in three individuals. In all patients, progression was not clearly stated. Instead, only symptoms of progression were described and not enough context information was present in the output data to determine the correct time of progression, causing a delay in detection of the time of progression. A ratio <0.33 was not observed.

## Discussion

In this study we explored the potential use of a rule-based TM approach to extract valuable data from the EMR. We explicitly wanted to develop a code using a limited number of functions from well-known R-packages. This may lower the threshold for clinical researchers to use this TM approach if they already use R for statistical analyses. This approach can significantly increase the efficiency of reliable data collection from EMRs compared to manual data collection. In addition, the inner workings of the developed systematic approach are accessible in contrast to those of commercial TM tools.

Recent advances in the field of artificial intelligence (AI) are rapidly reshaping cancer research, with applications in cancer diagnosis, clinical outcome prediction and drug discovery amongst others.^8^^,^^9^ However, current AI models within the field of oncology are heavily focused on imaging and omics data. In contrast, data from the EMR are vastly underutilized, which is mainly due to the intensive labor needed to collect the data from unstructured text.^8^ Therefore, TM approaches are essential tools to advance the development of AI models using EMR data.

In the study by Van Laar et al., a commercially available TM tool was compared to manual data collection. In this study, the TM tool missed a considerable amount of PS data, which was probably because the TM tool can only extract information meeting the exact search criteria.^5^ In our study, the use of pre-processing steps lowered the possible ways in which the PS could be written. In addition, the use of a single regular expression made it possible to search for multiple formats of PS at once. This probably enabled the detection of almost all PSs present in the EMR in our study.

The median PFS in the study by Van Laar et al. differed by ∼1 month, in which the TM tool detected the time of progression later than manual data collection. This is probably due to the use of suboptimal search queries as stated by the authors.^5^ In our study, the TM approach also tended to detect the time of progression later than manual data collection. However, the difference between the two methods was minimal. This is probably attributable to the use of n-grams to detect relevant strings of information instead of defining search terms based on expert knowledge only.

One of the main strengths of this study is the simplicity of the rule-based TM approach. In addition, the scripts were designed in a manner that can be transformed for purposes other than those described in this study. For example, the pre-processing steps and the regular expression in the PS script can be modified to obtain other measurements such as weight and blood pressure, if these data are frequently documented in an unstructured manner. In contrast, the PFS script is specifically designed to determine the date of progression. However, by reassigning the drug of interest and other treatments associated with progression, the date of progression can be determined for numerous treatments across different cancer types. Lastly, the regular expressions in the provided scripts can be changed to extract data from EMRs in different languages. This is facilitated by step 6 as relevant text strings are collected and used to define the regular expressions in step 7.

One of the main limitations of the rule-based approach used in this study is its inability to recognize text that is not exactly encoded. However, this can be partially overcome by screening the remaining data using n-grams to detect the remaining relevant strings of information. In addition, relevant strings of information may not be detected due to spelling mistakes as these were not taken into account. If a specific spelling mistake frequently occurs, it is possible to address this in the pre-processing step or during the step in which relevant regular expressions are defined. Furthermore, the TM approach has not been validated as a replacement for the gold standard of manual data collection. Rather than replacing it, TM approaches should be used before manual data extraction. Patients can then be roughly divided into three categories. The data extraction was successful, unsuccessful or there was some uncertainty in the reliability of the extracted data. In successful cases, no manual data collection is needed anymore. In the latter two cases, manual data collection should be carried out. Cases in which there was doubt in the determination of the progression date were often caused by sparse or vague descriptions. In addition, large time intervals between consecutive lines of information may also be a reason to manually determine the date of progression in the EMR. Because we wanted to compare the TM approach with manual data collection in this study, manual data collection from the EMR in case of doubt was not allowed, which caused some of the large differences between the TM and manually obtained PFS.

As research to facilitate data collection is still limited, it is of interest to determine whether statistical NLP systems can also be developed and used. These systems are able to learn from training data to detect things that may not necessarily be present in the training dataset.^4^ The field of AI for named-entity recognition is advancing and may become the gold standard for data collection in the future.^10^^,^^11^ Until this is the case, clinical researchers who have limited knowledge of AI and who currently carry out extensive manual data collection can use our TM approach to facilitate data collection.

In conclusion, we developed a TM approach to extract PS and PFS from the EMR with a very good performance. Therefore, this approach can significantly increase the efficiency of reliable data collection from EMRs by clinical researchers. In addition, this will facilitate the use of valuable RWD in research addressing clinical and policy-related questions.
