# Supplementary material for: Clinical text mining of the performance status and progression-free survival to facilitate data collection in cancer research: an exploratory study
Source: ESMO Real World Data Digit Oncol. 2024 Aug 13;5:100059. doi: 10.1016/j.esmorw.2024.100059 (PMC12836783; doi:10.1016/j.esmorw.2024.100059)
Supplement: Supplementary Tables [file mmc1.docx]

**Supplementary Tables**

**Table S1. Layout of the input files and description of the information documented in each column.**

| Column | Explanation of data |
| --- | --- |
| Patientid  *Patient identifier* | Number tracing back to the patient of which the data originates. |
| Lijsten  *Lists* | Textual path describing from what specific field in the electronic medical record the unstructured text is derived from. |
| LijstIDs  *List identifiers* | Numerical path corresponding to the textual paths of **lists**. |
| Stelling  *Question* | Name of the field in which the unstructured text is described under a **list**. |
| VraagID  Question identifier | Numerical name corresponding to the name of the **question**. |
| Datum  *Date* | Date on which unstructured text has been documented into the electronic medical record. |
| Xantwoord  *Anwser* | The unstructured data documented by a healthcare provider into the electronic medical record. This is the column from which data extraction is performed. |

**Table S2. Examples of regular expressions and the text strings that can be found.**

| Regular expressions | Detectable text strings |
| --- | --- |
| “(stop\|discontinue)\\s(the\\s)?osimertinib” | “stop osimertinib”  “stop the osimertinib”  “discontinue osimertinib”  “discontinue the osimertinib” |
| “(?<!osimer)tinib” | All tyrosine-kinase inhibitors except osimertinib: alectinib, crizotinib, erlotinib, gefitinib, trametinib, … |
| “no.{,20}progression” | “no progression”  “no clear progression”  “no tumor progression”  “no signs of progression” |

For a more elaborate explanation of regular expressions, see [1,2].

**References**

1. Regular expressions. [cited 2023 Apr 11]. Available from: https://cran.r-project.org/web/packages/stringr/vignettes/regular-expressions.html

2. Stringr. [cited 2024 Apr 15]. Available from: https://cran.r-project.org/web/packages/stringr/readme/README.html
