# Supplementary material for: Clinical text mining of the performance status and progression-free survival to facilitate data collection in cancer research: an exploratory study
Source: ESMO Real World Data Digit Oncol. 2024 Aug 13;5:100059. doi: 10.1016/j.esmorw.2024.100059 (PMC12836783; doi:10.1016/j.esmorw.2024.100059)
Supplement: Supplementary Pdf [file mmc2.pdf]

```
### --------------------------------------------------------------------------------------------------------------
### Clinical text mining of real world data to facilitate data collection
### in cancer research: an exploratory study
### Part 1: extraction of the performance status (PS)
### --------------------------------------------------------------------------------------------------------------

# load necessary packages
packages <- c("tidyverse", "readxl", "writexl")
lapply(packages, require, character.only = T)

# set working directory
setwd("_____")

# remove all objects in the environment
rm(list = ls())

# -----------------------------------------------------------------------------------------------------------------
### 1. Import the data set with unstructured data (data) and the data set with
### patientIDs and date of treatment start (osi)
data <- read_excel("_____.xlsx")
osi  <- read_excel("_____.xlsx")

```

```
# ---------------------------------------------------------------------------------------------------------------
### 2. Extract PS from structured data, if available
ps_structured <- data %>%
  filter(STELLING == "WHO performance status") %>%                     # filter the structured data rows in data
  mutate(number = str_sub(xantwoord, -1))                              # format of 'xantwoord' is 'VALUE1 = PS',
                                                                       # therefore, PS is the last character in the string
# ---------------------------------------------------------------------------------------------------------------
### 3. Text pre-processing to prepare the unstructured data for data extraction
data <- data %>%
  mutate(xantwoord = tolower(xantwoord),                               # change uppercase to lowercase
         xantwoord = str_remove_all(xantwoord, "[\\p{P}\\p{S}&&[^.,]]+"),   # remove all punctuation except ‘.’ And ‘,’
         xantwoord = str_remove_all(xantwoord, "[:space:]"),           # remove white spaces
         xantwoord = gsub("kps", "karnofsky", xantwoord, fixed = T))   # remove interference of 'kps'

# ---------------------------------------------------------------------------------------------------------------
### 7. Extract PS from unstructured data
ps_unstructured <- data %>%
  mutate(number = str_extract(xantwoord, "(who|ps|ecog|performancescore)\\d{1,2}"),   # regular expression to find PS in text
         number = gsub("\\D", "", number),                             # retain only numbers
         number = factor(number, levels = c("0", "1", "2", "3", "4",   # retain numbers mentioned within factor
                                            "01", "12", "23", "34"))) %>%   # as other numbers cannot be a PS
  filter(!is.na(number))                                               # remove rows without a detected PS
```

```
# -------------------------------------------------------------------------------------------------------------
### 8. Combine the PS data sets and match it to date of treatment start
ps <- ps_structured %>%
  bind_rows(ps_unstructured) %>%                                          # combining structured and unstructured PSes
  left_join(osi, by = c("patientid"))  %>%                                # match PS of each patient to ‘start_date’
  mutate(diff = as.numeric(as.Date(DATUM) - as.Date(start_date)))         # calculate time difference to use as a filter

# a time window of 30 days before until 14 days after treatment start was
# allowed for the matching
ps_before_start <- ps %>%
  filter(between(diff, -30, 0)) %>%                                       # up until 30 days before start
  group_by(patientid) %>%                                                 # within each patient
  filter(diff == max(diff)) %>%                                           # filter date and number closest to 'start_date'
  distinct(DATUM, number)                                                 # remove duplicates

ps_after_start <- ps %>%
  filter(between(diff, 1, 14)) %>%                                        # up until 14 days after start
  group_by(patientid) %>%
  filter(diff == min(diff)) %>%
  distinct(DATUM, number)
```

```
# PS before treatment start is preferred over one after treatment start
output <- osi %>%
  left_join(ps_before_start, by = c("patientid")) %>%                          # match closest 'ps_before_start' to 'start_date'
  left_join(ps_after_start, by = c("pitientid")) %>%                           # match closest 'ps_after_start' to 'start_date'
  mutate(ps = case_when(is.na(number.x) ~ number.y, T ~ number.x),             # use PS and date before 'start_date' if available
         date = case_when(is.na(DATUM.x) ~ DATUM.y, T ~ DATUM.x)) %>%          # otherwise, use PS and date after 'start_date'
  select(patientid, start_date, ps, date)                                      # select relevant rows for the output data

# -------------------------------------------------------------------------------------------------------------------
### Export created output data set
write_xlsx(output, "output.xlsx")

### end of code ##################################################################
```
