# Supplementary material for: Clinical text mining of the performance status and progression-free survival to facilitate data collection in cancer research: an exploratory study
Source: ESMO Real World Data Digit Oncol. 2024 Aug 13;5:100059. doi: 10.1016/j.esmorw.2024.100059 (PMC12836783; doi:10.1016/j.esmorw.2024.100059)
Supplement: Supplementary Pdf [file mmc3.pdf]

```r
### --------------------------------------------------------------------------------------------------------------
### Clinical text mining of real world data to facilitate data collection
### in cancer research: an exploratory study
### Part 2: extraction of progression free survival (PFS)
### --------------------------------------------------------------------------------------------------------------

# load necessary packages
packages <- c("tidyverse", "tidytext", "data.table", "readxl", "writexl")
lapply(packages, require, character.only = T)

# set working directory
setwd("_____")

# remove all objects in the environment
rm(list = ls())

# ------------------------------------------------------------------------------------------------------------------
### 1. Import data set with unstructured data (data) and
###    data set with patientID and date of treatment start (osi)
data <- read_excel("_____.xlsx")
osi  <- read_excel("_____.xlsx")

```

```
### remove unstructured data rows before date of treatment start,
### since these rows are not needed
data <- data %>%
  mutate(xantwoord = tolower(xantwoord),                              # change uppercase to lowercase
         xantwoord = gsub("?", "??", xantwoord, fixed = T)) %>%       # retain "?" after tokenization
  left_join(osi, by = c("patientid")) %>%                             # add date of treatment start to each row
  filter(DATUM >= start_date) %>%                                     # remove rows with date before treatment start
  select(patientid, STELLING, start_date, xantwoord, DATUM)           # select columns to work with

### ------------------------------------------------------------------------------------------------------------
### 2. Extract PFS from structured data, if available (only possible answers
###    in 'xantwoord' are 'remission' and 'progression')
result_ow <- data %>%
  filter(STELLING == "Ontwikkeling ziekte") %>%                       # filter the structured data rows
  mutate(s = if_else(xantwoord == "remissie", "remissie", "progressie"),  # make column 's' containing separate sentences
         drug = NA,                                                   # output columns are defined
         bp = if_else(s == "remissie", "yes", "n"),                   # bp: before progression
         pp = if_else(s == "progressie", "yes", "n"),                 # pp: possible progression
         ap = "n",                                                    # ap: after progression
         rb = "100%")                                                 # rb: reliability


```

```
### -----------------------------------------------------------------------------------------------------------------------
### 3. Text pre-processing to prepare the unstructured data for data extraction
d_bco <- data %>%
  filter(STELLING != "Ontwikkeling ziekte") %>%                                 # filter the unstructured data rows
  mutate(xantwoord = str_replace_all(xantwoord,
          c("(?<!\\w)(de|het)(?!\\w)|\\s+"       = " ",                        # remove 'de' and 'het' and multiple white spaces
            "(?<=[[:lower:]])\\.(?=[[:lower:]])"  = "",                         # remove interference from abbreviations
            "(?<!\\w)dd\\."                      = "differentiaal\\sdiagnose",  # change 'dd.' to 'differential diagnosis'
            "(?<!\\w)dr\\.|(?<!\\w)prof\\."      = "dokter",                    # change 'dr.' and 'prof.' to 'doctor'
            "(?<!\\w)osi(?!\\w)"                 = "osimertinib",               # change 'osi' to 'osimertinib'
            "(?<!\\w)crizo(?!\\w)"               = "crizotinib")))              # change 'crizo' to 'crizotinib'

# in the original script this part is a bit longer in which other interfering
# abbreviations were also removed

### ----------------------------------------------------------------------------
### 4. Define the drug of interest (drug) and other treatments (f_ot)              # assigning these values makes changing the
###  that can be mentioned in case of progression                                  # script for other drugs easier and shortens
drug <- "osimertinib|tagrisso"                                                     # the code by avoiding repetition of all drugs
f_ot <- "(?<!osimer)tinib|chemo|carbo|platin|taxol|taxel|pacli|pemetrexed|umab"    # in f_ot


```

```r
# custom made function to add output columns
# only variables that deviate from the default values need to be defined
c_mutate <- function(df, bp = "n", pp = "n", ap = "n", rb = "high") {
  df_output <- df %>%
    mutate(bp = bp, pp = pp, ap = ap, rb = rb)
  return(df_output)
}

### ------------------------------------------------------------------------------------------------------------------------
### 5. Unnest tokens: split text into separate sentences
d_bco <- d_bco %>%
   unnest_tokens(input = xantwoord, output = "s", token = "regex",
       pattern = "(?<!\\d)\\.(?=\\s)|(\\!|\\?|;)(?=\\s)|\\s{4,}|\r\r\n", drop = F) %>%
   filter(grepl("[[:alnum:]]+", s))

### ------------------------------------------------------------------------------------------------------------------------
### 6. Use n-grams to detect relevant strings of information in the unstructured
### data indicating BP, PP and AP
freq_ngram <- d_bco %>%                                                # 'n = 3' can be changed to look for a different
   unnest_tokens(ngram, s, token = "ngrams", n = 3, drop = F) %>%      # number of successive words
   count(ngram, sort = T)
```

```
### ---------------------------------------------------------------------------------------------------------------------
### 7. Select rows containing relevant information using regular expressions
###    and create output columns.

# make column for the presence of the  drug of interest in the sentence
d_bco$drug <- ifelse(grepl(drug, d_bco$s), "mentioned", NA)

# strings of text indicating BP and rb = high
so_bp_high <- d_bco %>%
  filter(grepl("start|switch", s) & grepl(drug, s) |
        # (start|switch) in the presence of 'osimertinib
      (grepl("continue|goed\\s(verdragen|tijdens)|remissie|zo\\sdoor|doorgaan", s, perl = T) & !grepl("progressie|geen", s)) |
        # (continue|well-tolerated|remission) in the absence of the words (progression|no)
      (grepl("(?<!\\w)(sd|pr|cr)(?!\\w)|(stabiele\\sziekte|partiele\\srespons|complete\\srespons)", s, perl = T) & !grepl("geen|niet", s)) |
        # (sd|pr|cr|stable disease|partial response|complete response) in the absence of the words (no|none)
      (grepl("(goede|mooie|prachtige|fraaie|ongoing|aanhoudende|persisterende)\\s(respons|resultaat|remissie)", s)) ) %>%
        # (good|beautiful|ongoing|persistent) followed by (response|result|remission)
  filter(!grepl(f_ot, s, perl = T)) %>%
   # remove rows containing treatments mentioned in 'f_ot'
  c_mutate(bp = "yes")
```

```
# strings of text indicating BP and rb = medium
so_bp_medium <- d_bco %>%
  anti_join(so_bp_high, by = c("patientid", "STELLING", "start_date", "xantwoord", "DATUM", "s")) %>%
  filter(grepl("(?<!mixed\\s)respons|resultaat", s, perl = T) & !grepl(f_ot, s, perl = T)) %>%
  c_mutate(bp = "yes", rb = "medium")

# strings of text indicating BP but potentially close to PP
so_bppp_high <- d_bco %>%
  filter(grepl("mixed\\srespons", s)  |                                   # mixed response
      (grepl("progressie", s) & grepl("\\?", s)) ) %>%                     # progression combined with question mark
  filter(!grepl(f_ot, s, perl = T)) %>%
  c_mutate(bp = "yes", pp = "yes")

# strings of text indication PP and rb = high
so_pp_high <- d_bco %>%
 filter((grepl("(wegens|ivm|evidente|sprake\\svan)?\\s?progressie", s, perl = T) & !grepl("geen", s) & !grepl(f_ot, s, perl = T)) |
       # (because of|due) followed by 'progression', in the absence of 'no' and f_ot
      (grepl("progressie.{0,10}ziekte", s) & !grepl("geen.*progressie", s)) |
       # 'progression' followed by max 10 characters and then followed by 'disease', in which 'no' is not allowed before progression
      (grepl("(?<!\\w)pd(?!\\w)", s, perl = T) & !grepl("geen", s) & !grepl("pd-l1", s) & !grepl(f_ot, s, perl = T)) )%>%
       # 'pd' in the absence of 'no', 'pd-l1' and f_ot
 c_mutate(pp = "yes")
```

```
# strings of text indicating AP and rb = high
so_ap_high <- d_bco %>%
  filter((grepl("start\\scrizotinib", s) & grepl(drug, s)) |
         # start a targeted therapy ending with 'tinib' in combination with osimertinib
        (grepl("(?<!\\w)(pd|sd|pr|cr)(?!\\w)|(progressieve\\s|stabiele\\s)ziekte|(partiele\\s|complete\\s)respons", s, perl = T)
        & grepl(f_ot, s, perl = T)) |
         # (sd|pr|cr|stable disease|partial response|complete response) in the presence of treatment mentioned in f_ot
        (grepl("switch\\s|start", s) & grepl(f_ot, s, perl = T) & !grepl(drug, s)) ) %>%
         # start or switch to treatment mentioned in f_ot in the absence of the word osimertinib
   c_mutate(ap = "yes")

### combining the separate data sets and remove the separate data sets from the environment
dfs <- lapply(ls(pattern = "^so_"), function(x) get(x))
result_bco <- rbindlist(dfs, fill = T)
rm(list = ls(pattern = "^so_"))

### ---------------------------------------------------------------------------------------------------------------------
### 8. Combine the structured and unstructured data sets and match date of CT
###    or MRI scan to the date of outpatient visit
result <- bind_rows(result_ow, result_bco)
```

```
# the code matching scan_date is not shown as it is too specific for our institute
# due to data migration and the use of different date formats, this part of the code
# is not understandable without the input data itself.
# however, a similar code as shown in the PS script can be used to match the scan_date
# to the corresponding date of outpatient visit

### ---------------------------------------------------------------------------------------------------------------
### 9. Results are organized in order of patientID and date

# remove rows describing progression on start_date, as these often describe progression
# on the treatment that was given before osimertinib was initiated
result_remove <- result %>% filter(start_date == DATUM, pp == "yes" |ap == "yes")

result <- result %>%
  anti_join(result_remove) %>%
  arrange(patientid, DATUM) %>%
  distinct(patientid, xantwoord, DATUM, s, drug, bp, pp, ap, rb, .keep_all = T)

# data can be either exported or viewed within R to determine the date
# of progression or censoring

### end of code ######################################################################
```
